# Retraction: Bee venom-loaded EGFR-targeting peptide-coupled chitosan nanoparticles for effective therapy of hepatocellular carcinoma by inhibiting EGFR-mediated MEK/ERK pathway

**DOI:** 10.1371/journal.pone.0287805

**Published:** 2023-06-23

**Authors:** The PLOS ONE Editors

Following the publication of this article [1], concerns were raised regarding multiple figures. Specifically,

- In Fig 7B, clusters of cells within the HepG2 non-treated well appear similar to each other, and to clusters of cells in the SMMC-7721 non-treated well.
- In S1 Raw images, the original blots underlying the following pairs of total and phosphorylated blots appear to have similarities in marker bands and background pattern:
  ○ MEK and p-MEK
  ○ ERK and p-ERK
- In the Bcl2 image in S1 Raw images, there appears to be a discontinuity in the background between the marker lane and lane 1.
- In the p-p38MAPK image in S1 Raw images, the band in lane 1 appears to overlap with the marker lane.
- The SMMC-7721 cell line reported in this study as a human liver cancer has been identified as a contaminated cell line, and was shown to be a HeLa derivative cell line [2, 3] prior to the publication of this article [1].

The corresponding author stated that all data underlying the results in this article are available, except for raw data underlying the flow cytometry experiment in Fig 8.

The corresponding author acknowledged that clusters of cells in two wells in Fig 7B appear similar, and they stated that the similarity was observed in other experiments using these cells. The *PLOS ONE* editors remain concerned regarding similarity of cells within and between wells.

For pairs of total and phosphorylated proteins (MEK and ERK), the corresponding author clarified that blots were not stripped and reprobed. They stated that total and phosphorylated protein were run on the same gel, blots were cut prior to incubation with antibodies, then imaged independently using the same marker lane. They provided different versions of the original blots without the marker lane added. The editors remain concerned about the similarities between the images for total and phosphorylated blots, in particular the similarities in background pattern.

The corresponding author’s responses regarding the Bcl2 and p-p38MAPK images in S1 Raw images did not resolve the concerns. They also stated that the SMMC-7721 cells used for this study were not authenticated prior to use.

In light of the concerns affecting multiple figure panels that question the integrity of these data, the *PLOS ONE* Editors retract this article.

MB did not agree with the retraction. SA, NM, MG, MEK, and AIE either did not respond directly or could not be reached.
